# Giant baleen whales emerged from a cold southern cradle

**DOI:** 10.1098/rspb.2023.2177

**Published:** 2023-12-20

**Authors:** James P. Rule, Ruairidh J. Duncan, Felix G. Marx, Tahlia I. Pollock, Alistair R. Evans, Erich M.G. Fitzgerald

**Affiliations:** ^1^ School of Biological Sciences, Monash University, Melbourne, Victoria 3800, Australia; ^2^ Sciences, Museums Victoria Research Institute, Museums Victoria, Melbourne, Victoria 3001, Australia; ^3^ Department of Life Sciences, Natural History Museum, London SW7 5BD, UK; ^4^ Museum of New Zealand Te Papa Tongarewa, Wellington 6011, New Zealand; ^5^ Department of Geology, University of Otago, Dunedin 9016, New Zealand; ^6^ Department of Anatomy and Developmental Biology, Monash University, Victoria, Australia; ^7^ National Museum of Natural History, Smithsonian Institution, Washington, DC 20013, USA

**Keywords:** Mysticeti, Chaeomysticeti, Southern Hemisphere, body size, gigantism

## Abstract

Baleen whales (mysticetes) include the largest animals on the Earth. How they achieved such gigantic sizes remains debated, with previous research focusing primarily on when mysticetes became large, rather than where. Here, we describe an edentulous baleen whale fossil (21.12–16.39 mega annum (Ma)) from South Australia. With an estimated body length of 9 m, it is the largest mysticete from the Early Miocene. Analysing body size through time shows that ancient baleen whales from the Southern Hemisphere were larger than their northern counterparts. This pattern seemingly persists for much of the Cenozoic, even though southern specimens contribute only 19% to the global mysticete fossil record. Our findings contrast with previous ideas of a single abrupt shift towards larger size during the Plio-Pleistocene, which we here interpret as a glacially driven Northern Hemisphere phenomenon. Our results highlight the importance of incorporating Southern Hemisphere fossils into macroevolutionary patterns, especially in light of the high productivity of Southern Ocean environments.

## 1. Introduction

Gigantic baleen whales embody the extreme upper end of body size evolution. Previous studies have attributed this pattern to two separate processes, namely, (i) the sudden disappearance of most small mysticetes around 3 Ma [1,2] and (ii) the evolution of gigantism itself. The origin of their large size remains debated [1,3,4], with some studies proposing an abrupt mode-shift towards larger size that coincided with the Pliocene extinction of smaller species and was driven by seasonal, localized upwelling patterns [1,5–8], whereas others argue for earlier evolutionary rate shifts and/or a more gradual increase in maximum body size through time [9–11].

Some toothed mysticetes attained body lengths of 8–12 m relatively early, prior to their disappearance around 23 Ma [3,9,12]. By contrast, edentulous baleen whales (chaeomysticetes) seemingly did not exceed 8 m until about 10 Ma [1]. Confirming this pattern is, however, complicated by a global Early Miocene gap (or ‘dark age') in the mysticete fossil record [13,14], as well as strong collection and rock-record biases against much of the Southern Hemisphere [6,15,16].

Here, we report a large chaeomysticete fossil from the Early Miocene of South Australia and revisit the early evolution of baleen whale body size with a particular focus on the Southern Hemisphere.

## 2. Methods

**Figure 1.**
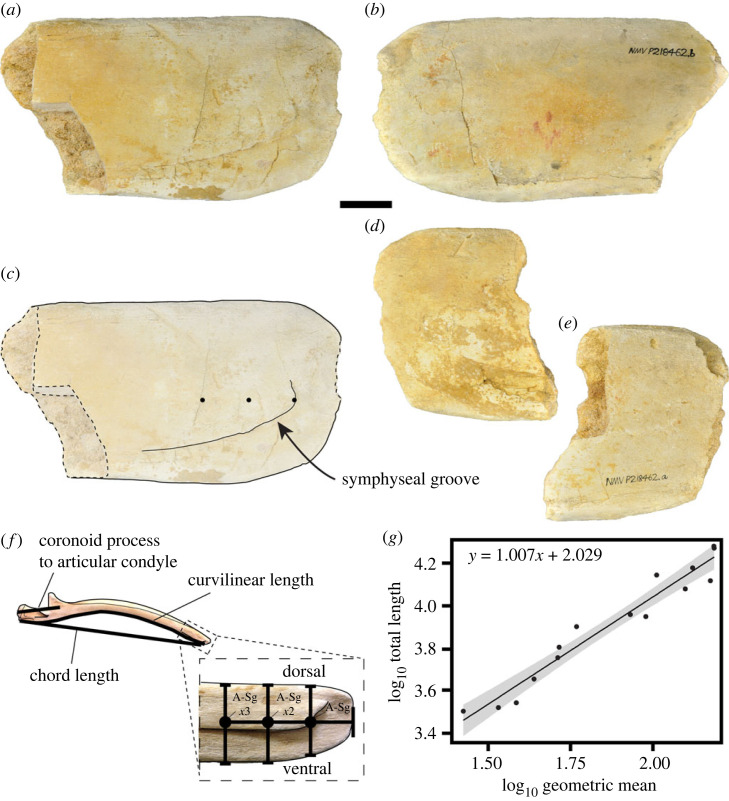
Chaeomysticete mandible fragments NMV P218462 (Museums Victoria) from the Aquitanian–Burdigalian of South Australia. Left mandible apex in (*a*) medial and (*b*) lateral views, and annotated medial view (*c*) with symphyseal groove and measurement landmarks highlighted. Right mandibular apex in (*d*) medial and (*e*) lateral views. Measurements taken from mandibles (*f*) for estimation of total body length (NMV C24936 *Balaenoptera acutorostrata* pictured). Resulting regression (*g*) of log_10_ total body length and log_10_ geometric mean of the mandible apex for estimating the total length of NMV P218462. Scale bar equals 50 mm.

To estimate the total length of our new fossil, we first generated predictive regression equations based on five mandibular measurements from seven extant mysticete species (figure 1; see electronic supplementary material for details and data). All measurements were log_10_ transformed prior to analysis. The best-performing equation (figure 1*g* and electronic supplementary material, table S3) was selected using per cent prediction error and per cent standard error of the estimate (electronic supplementary material).

To investigate mysticete size evolution in a broader macroevolutionary context, we assembled a comprehensive total body length dataset covering living and extinct species (building on [1]) and paired it with a composite phylogeny built from multiple sources [1,10,17] in Mesquite version 3.7 [18]. Subsequent analyses were run in R-studio version 2022.07.1 using R version 4.2.1 [19].

We visualized geographical differences in body size distribution via a scatterplot of mysticete body length through time with separate polynomial trendlines for Northern and Southern Hemisphere taxa (via ggplot2; [20]). To assess the effect of interhemispheric sampling and rock-record biases, baleen whale fossil occurrence data were downloaded from the Paleobiology database [21]. Finally, we generated hemisphere-specific maximum-likelihood ancestral state estimations and lineage-through-time plots using geiger and phytools [22,23].

To trace the deep-time evolution of mysticete body length, we plotted the size categories of [4] on the composite phylogeny using maximum-likelihood ancestral state estimations and then generated attendant lineage-through-time plots. Next, we used RRphylo [24] to perform maximum-likelihood ancestral state estimations and rate shift analyses of two transformations of total body length, namely, log_10_ and generation time, following [25]. We used the *auto.recognize* feature to identify rate shifts, and *overfitRR* to test for uncertainty in the rate shifts across nodes.

## 3. Results

### (a) Systematic palaeontology

Cetacea Brisson 1762 [26]

Neoceti Fordyce & Muizon 2001 [27]

Mysticeti Gray 1864 [28]

Chaeomysticeti Mitchell 1989 [29]

Chaeomysticeti gen. et sp. indet.

*Referred specimen*. NMV P218462, symphyseal regions of both mandibles, plus fragments of the left premaxilla and maxilla.

*Locality and horizon*. NMV P218462 was found by F.A. Cudmore (on 15 February 1921) eroding from limestone cliffs on the east bank of the Murray River, opposite Wongulla, about 5 km south of Devon Downs, South Australia. Measured sections of cliff outcrop both north (Devon Downs) and south (Walker Flat) of Wongulla show that the only unit forming the continuous cliffs along this stretch of river is the Mannum Formation [30]. The latter is divided into a lower (Late Oligocene) and upper (Early Miocene) unit [31]. Matrix associated with NMV P218462 is limonite-stained yellow-brown coloured, fine-grained calcarenite. This lithology is characteristic of the upper Mannum Formation [30], which based on planktonic foraminifera is assigned to the M2–M4 Planktonic Foraminiferal Zones [31], within the Aquitanian–Burdigalian (21.12–16.39 Ma) [32].

*Comments on systematic attribution.* NMV P218462 is referable to Kinetomenta (*sensu* [33]) based on the presence of an unsutured symphysis and distinct symphyseal groove; and to Chaeomysticeti based on the lack of mandibular alveoli, the presence of multiple relictual alveolar foramina with associated anterior sulci, and the relatively small height and dorsal placement of the mandibular canal.

### (b) Body size and biogeographic analyses

**Figure 2.**
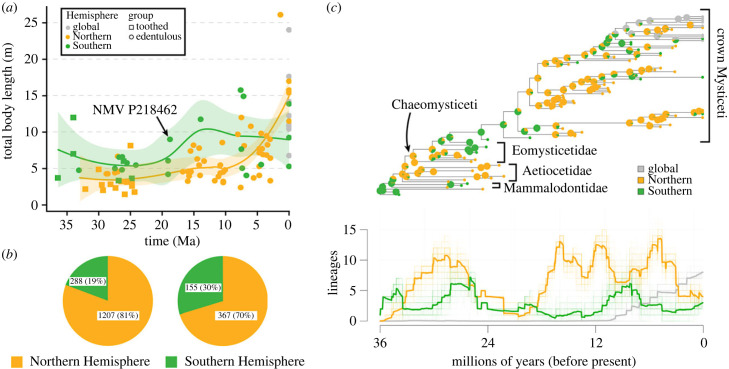
Biogeography of Mysticeti. Total body length (in metres) plotted against time (*a*) for both toothed and edentulous baleen whales from the Northern and Southern Hemispheres (median stratigraphic age for fossils from [2]); local polynomial regressions demonstrate differences in size between the two Hemispheres. Fossil occurrence data for Mysticeti (*b*) downloaded from the Palaeobiology database [21] demonstrating the sampling bias (left) and number of formations (right) between hemispheres. All-rates-different model maximum-likelihood ancestral state estimation (*c*) of biogeographic region for mysticetes, with a lineage-through-time plot.

The best-performing regression equations use a geometric mean of measurements from the anterior end of the mandible (figure 1*g*; electronic supplementary material, table S3) and estimate NMV P218462 to be approximately 9 m long. A scatterplot of total body length against time shows that Southern Hemisphere mysticetes, including NMV P218462, tend to be larger than their northern counterparts, even though southern fossils only contribute 19% to the global sample (figure 2*a,b*). The best-supported ancestral state estimation model (all-rates-different) and corresponding lineage-through-time plot show that Southern Hemisphere lineages are underrepresented across the entire phylogeny (figure 2*c*).

**Figure 3.**
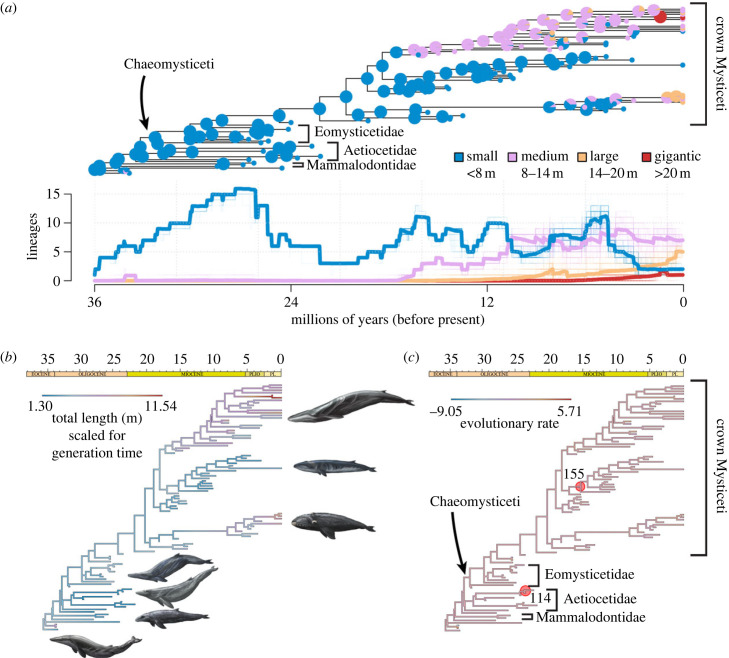
Phylogenetic distribution of total body length through time for toothed and edentulous baleen whales. Symmetrical model maximum-likelihood ancestral state estimation (*a*) of (qualitative) total body length as categorical data (categories from [4]) and resulting lineage-through-time plot. Maximum-likelihood ancestral state estimation (*b*) and evolutionary rates (*c*) of (quantitative) total body length scaled to generation time. Red circles indicate a (statistically significant) decrease in evolutionary rate across the labelled node. Whale illustrations by R.J.D.

Ancestral state estimations of total body length show some variation across the phylogeny when log_10_ transformed (electronic supplementary material, figure S2), but change more gradually when grouped into size categories (symmetrical model, figure 3*a*) or scaled to account for generation time (figure 3*b*). Mysticetes were mostly small for the first half of their evolutionary history and then gradually became larger after approximately 16 Ma (figure 3*a*,*b*). Small baleen whales abruptly decline between 4 and 3 Ma. Evolutionary rates remain relatively steady and show few shifts irrespective of the type of transformation applied to the data (figure 3*c*; electronic supplementary material, figure S3; table S5). Two shifts occur in both analyses (figure 3*c*; electronic supplementary material, figure S3 and table 1): an increase in evolutionary rate across node 101, near the root (*p* = 1.00, with *p* > 0.975 reflecting a significant increase); and a decrease across node 114 inside the stem family Aetiocetidae (*p* < 0.05, detected in over 90% of overfit simulations).

**Table 1.** Results of search.shift analysis and associated overfit values for log_10_ total body length (root value: 0.767; 2.5% CI: 0.755; 97.5% CI: 0.861) and total body length scaled for Generation Time (GT) (root value: 4.087; 2.5% CI: 4.054; 97.5% CI: 4.779). Body length data in metres prior to transformation. Rate shifts are identified if *p* > 0.975 or *p* < 0.025.

	clade	rate difference	*p*-value	positive shift	negative shift
log_10_	all clades	–	–	0.43	0
	114	−0.039	0.007	0	0.91
	193	−0.033	0.021	0.04	0.12
	101	0.413	1	0.52	0
GT	all clades	–	–	0.13	0.24
	114	−0.315	0.001	0	0.96
	155	−0.254	0.001	0	0.87
	101	3.76	1	0.58	0

## 4. Discussion

### (a) Large southern mysticetes and sampling bias

Previous research sought to date the origin of gigantism in mysticetes [3–6,10] but paid relatively little attention to its geographical origin. This is potentially problematic, as the where is likely to bear on the why. At roughly 9 m long, NMV P218462 is the largest baleen whale recorded from the Early Miocene, and indeed the oldest medium-sized chaeomysticete worldwide. Along with other large Middle–Late Miocene specimens from Peru [10], it suggests a more gradual—albeit hemisphere-specific—emergence of mysticete gigantism than previously thought [1,5–7].

Both toothed and edentulous mysticetes seemingly evolved larger earlier in the Southern Hemisphere, even though southern fossils remain heavily undersampled (figure 2). Rather than abruptly increasing in size during the Plio-Pleistocene [1,6], southern species achieved relatively large sizes early on, perhaps as a result of greater regional productivity. Collection and rock-record biases (figure 2*b*, [34,35]) have largely obscured this pattern until now, despite the presence of promising localities across South America [36–40], south-western Africa [41–43], Australia [44] and New Zealand [45,46]. Crucially, austral sites have also shown some tentative promise in elucidating the Early Miocene gap in the global mysticete fossil record [13,14], which northern localities have failed to address despite centuries of research. Our results call for far greater sampling of the Southern Hemisphere to generate a truly global assessment of mysticete palaeodiversity and evolution.

### (b) Trends in mysticete body size evolution

Current data suggest that medium-large baleen whales only became common around 16 Ma (figure 3*a*). This idea needs to be interpreted with caution, however, as more informative finds from the Southern Hemisphere and, in particular, the Early Miocene baleen whale gap could plausibly push the rise of large-bodied species back in time. Perhaps a more reliable pattern—albeit again with the caveat of further southern discoveries—is the decline of small mysticetes starting around 4 Ma, triggered by more patchy resource distributions and/or the disrupting effects of Northern Hemisphere glaciation [1,2].

Limited variation in our ancestral state estimates and the low number of significant rate shifts suggest a relatively gradual emergence of large body size [10]. This is especially true when data are scaled to generation time (figure 3*b*), which is thought to be more accurate than traditional log-transformations in this context [25]. A glacially driven Plio-Pleistocene mode-shift towards gigantism remains possible [1,5–7], but seemingly builds on a more gradual, longer-term trend.

### (c) Productivity and gigantism

The Southern Ocean is crucial to modern mysticetes, both because of its high seasonal productivity and the contribution of the Antarctic Circumpolar Current (ACC) to global nutrient availability [47,48]. It is thus perhaps no coincidence that both toothed and edentulous baleen whales first evolved larger sizes in the south, with the earliest large-bodied species coinciding with the establishment of a proto-ACC, Antarctic glaciation and seasonally productive polar ecosystems around 36–33 Ma [49,50]. Further size increases were perhaps driven by the strengthening of the ACC [51,52] and the emergence of potential prey (e.g. krill) [53,54] during the Miocene. These interhemispheric differences were plausibly sharpened by warm equatorial waters acting as a barrier to dispersal, as seen among many extant mysticetes today [55].

Living mysticetes perform crucial roles as ecosystem engineers [56], e.g. by enhancing primary productivity via nutrient recycling [48,57–60]. For example, mysticetes may have catalysed 10–20% of Southern Ocean net primary productivity prior to whaling [48]. Effects of this magnitude are largely driven by modern blue and fin whales with extremely large body sizes, which correlate with increased feeding efficiency [61]. Nevertheless, smaller animals like NMV P218462, which is comparable in size to living minke whales, could have had an impact if population sizes were sufficiently large. If so, mysticetes may have helped to engineer ocean ecosystems for much of the Neogene and perhaps created a positive feedback loop where whale-enhanced productivity in turn sustained more whales. The same might also have been true of other large cetaceans like certain physeteroids, ziphiids and archaeocetes [11,62,63].

## 5. Conclusion

Baleen whales first evolved large body size in the Southern Hemisphere, perhaps facilitated by the onset of the ACC and high seasonal productivity in the Southern Ocean. Previous suggestions of an abrupt global Plio-Pleistocene shift towards mysticete gigantism are hampered by a strong collection bias against austral localities that obscures more gradual and regional trends. Medium-large mysticetes may have helped to engineer ocean ecosystems, albeit in a comparatively limited fashion, since the beginning of the Neogene. Further exploration of the Southern Hemisphere is crucial to constructing a truly global picture of the nature, timing and impacts of whale evolution.

## Data Availability

Supporting data for this publication can be found at Figshare (doi:10.6084/m9.figshare.20523216) [64]. The fossil described in this paper is lodged in a publicly accessible institution. Supplementary material is available online [65].
